# Steep-Slope and Hysteresis-Free MoS_2_ Negative-Capacitance Transistors Using Single HfZrAlO Layer as Gate Dielectric

**DOI:** 10.3390/nano12244352

**Published:** 2022-12-07

**Authors:** Xinge Tao, Lu Liu, Jingping Xu

**Affiliations:** School of Optical and Electronic Information, Huazhong University of Science and Technology, Wuhan 430074, China

**Keywords:** hafnium zirconium aluminum oxide (HZAO), negative-capacitance field-effect transistors (NCFETs), sub-threshold swing (*SS*), hysteresis, molybdenum disulfide (MoS_2_)

## Abstract

An effective way to reduce the power consumption of an integrated circuit is to introduce negative capacitance (NC) into the gate stack. Usually, negative-capacitance field-effect transistors (NCFETs) use both a negative-capacitance layer and a positive-capacitance layer as the stack gate, which is not conductive to the scaling down of devices. In this study, a steep-slope and hysteresis-free MoS_2_ NCFET is fabricated using a single Hf_0.5−x_Zr_0.5−x_Al_2x_O_y_ (HZAO) layer as the gate dielectric. By incorporating several Al atoms into the Hf_0.5_Zr_0.5_O_2_ (HZO) thin film, negative capacitance and positive capacitance can be achieved simultaneously in the HZAO thin film and good capacitance matching can be achieved. This results in excellent electrical performance of the relevant NCFETs, including a low sub-threshold swing of 22.3 mV/dec over almost four orders of drain-current magnitude, almost hysteresis-free, and a high on/off current ratio of 9.4 × 10^6^. Therefore, using a single HZAO layer as the gate dielectric has significant potential in the fabrication of high-performance and low-power dissipation NCFETs compared to conventional HZO/Al_2_O_3_ stack gates.

## 1. Introduction

As the size of MOSFETs continues to decrease, it has become increasingly difficult to reduce the size of the device below 10 nm. One reason for this is that the power consumption of the device is difficult to reduce. In traditional MOSFETs, the sub-threshold swing (*SS*) of the device is limited by the Boltzmann limit, resulting in the chip power consumption failing to reach the expected target [1,2,3,4,5]. To reduce the device power consumption, several novel device models have been proposed to achieve a sub-threshold swing (*SS*) lower than 60 mV/dec at room temperature. The first type proposed is the nano-electromechanical switch (NEM) [6,7]. The NEM is small and low in power consumption. Its *SS* can even reach single digits, to much lower than 60 mV/dec. However, the reliability of NEM devices is relatively low and the lack of expandable preparation technology is not conducive to large-scale process production. Another low-power device model is the tunnel field-effect transistor (TFET) [8,9,10,11]. In 2017, Adrian Jonescu proposed using the quantum tunneling effect to make TFETs. TFETs can significantly reduce the driving voltage imposed by the gate and have a very low turn-off current. Their *SS* can be lower than 60 mV/dec, with greatly reduced power consumption. However, the turn-on current is relatively low and the choice of materials, and the presence of defects in these, significantly impact the development of TFETs. The negative-capacitance field-effect transistor (NCFET) is considered an ideal choice for ultra-low power applications [1,2,4,12,13,14]. It only requires addition of a ferroelectric material as an additional layer to the gate dielectric layer of a traditional MOSFET to act as a negative capacitor to achieve voltage amplification of the channel surface potential, thus breaking the Boltzmann limit such that an *SS* less than 60 mV/dec can be achieved. At the same time, materials with high dielectric constants, such as HfO_2_ and ZrO_2_, having much higher dielectric constants than SiO_2_, wide band gaps and good thermal stability, are considered excellent gate dielectric materials and have been applied in process mass production. However, in Germany, Johannes Muller et al. reported that ferroelectric properties would appear when different elements, such as Si, Y, Al and Zr, were doped into HfO_2_ thin films, [15,16,17]. This discovery resulted in ferroelectric materials and NCFET becoming an international research craze [18,19,20,21]. Using HZO thin film as the ferroelectric layer, HZO NCFETs have become one of the solutions to achieving ultra-low-power CMOS technology. HZO NCFETs have the following advantages: (1) high conduction current; (2) symmetrical circuit layout; and (3) CMOS process compatibility. However, these NCFETs contain at least two layers as the gate stack, which complicates the fabrication process and is not conducive to the scaling down of devices.

In this investigation, a single-layer (HfZr)_0.5−0.5x_Al_x_O_y_ (HZAO) thin film was found to have both negative capacitance and positive capacitance due to its ferroelectricity, and, thus, capacitance matching could be realized by itself. A single HZAO layer was used as the gate dielectric to fabricate the relevant MoS_2_ NCFETs. By optimizing the Al content and the annealing temperature of the HZAO thin film, excellent electrical properties for NCFETs have been achieved, including a low *SS* of 22.3 mV/dec over almost four orders of drain-current magnitude, almost no hysteresis, and a high on/off current ratio of 9.4 × 10^7^, which, so far, is better than that achieved with HZO-based MoS_2_ NCFETs under the same fabrication and measurement conditions.

## 2. Experimental Method

Heavily-doped p^++^-Si wafers with a resistivity of 0.005~0.01 Ω·cm were cleaned by a standard RCA method as substrate/back gate and then were placed in an atom-layer deposition (ALD) chamber. As the ferroelectricity of the HZAO thin film is highly dependent on its annealing temperature and atom ratio [19,22,23], HZAO thin films with different atom ratios were prepared through alternately ALD-depositing Al_2_O_3_, HfO_2_ and ZrO_2_ at ratios of Al_2_O_3_:HfO_2_:ZrO_2_ = 1:5:5 or 1:10:10 or 1:20:20 or 0:1:1 to yield a nano-laminate Hf_0.4_Zr_0.4_Al_0.2_O_y_ or Hf_0.45_Zr_0.45_Al_0.1_Oy or Hf_0.475_Zr_0.475_Al_0.05_Oy or Hf_0.5_Zr_0.5_O_y_ (HZO) thin film on the p^++^-Si substrate using trimethylaluminium (TMA) as the Al source, tetrakis (ethylmethylamino)-Hf (TDMAH) as the Hf source, tetrakis (ethylmethylamino)-Zr (TDMAZ) as the Zr source, and H_2_O as the oxidant. During deposition, the temperatures of the substrate and the Hf/Zr sources were 200 °C and 75 °C, respectively. The resultant thicknesses of the thin films were 8.03 nm, 8.01 nm, 8.03 nm, and 8.00 nm, respectively, measured by ellipsometry. The thin films were then annealed by rapid thermal processing (RTP) at 600 °C, 650 °C, 700 °C and 750 °C, respectively, for a duration of 30 s.

MoS_2_ flakes were transferred from bulk crystal onto the HZAO or HZO/p^++^-Si substrates by a micromechanical exfoliating method using 3M tapes and PDMS films [24]. Electron-beam lithography (EBL) was used to pattern the source (S) and drain (D) electrodes of the transistors, followed by deposition of 15 nm Cr and 45-nm Au at room temperature by thermal evaporation and a lift-off process to form the S/D electrodes [25,26,27]. The drawn channel length (L) of these transistors was 3 μm and the channel width (W) was 3~5 μm, based on the shape of the flakes. Lastly, the transistors were annealed at 300 °C for 180 s in a N_2_ environment to improve the electrical contact between the MoS_2_ and metal electrode and to remove the gas and liquid molecules on the MoS_2_ [28].

**Figure 1:**
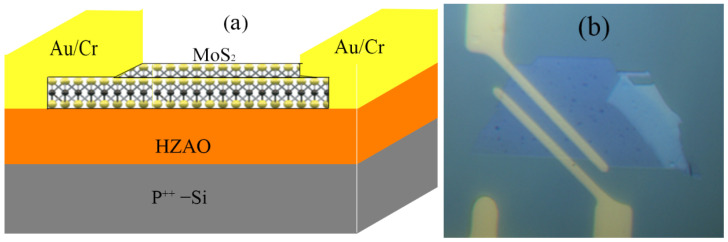
(**a**) Schematic diagram and (**b**) optical micrograph of a back-gated HZAO NCFET.

A schematic diagram of a back-gated NCFET is shown in Figure 1a and its optical micrograph is shown in Figure 1b.

## 3. Results and Discussion

### 3.1. Optimization of Al Content in HZAO Thin Film

**Figure 2:**
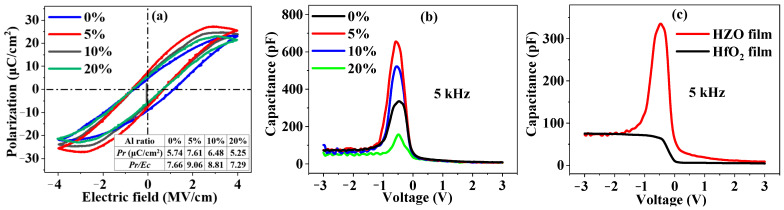
(**a**) *P-E* curves of HZAO thin films with different Al contents; (**b**) *C-V* curves of (Au/Cr)/HZAO/p^++^-Si capacitors with different Al contents at 5 kHz (annealing temperature: 650 °C); (**c**) *C-V* curves of (Au/Cr)/HZO/p^++^-Si and (Au/Cr)/HfO_2_/p^++^-Si and capacitors at 5 kHz (annealing temperature: 650 °C).

The polarization vs. field (*P-E*) measurements were carried out on the (Au/Cr)/HZAO/p^++^-Si capacitor to characterize the ferroelectricity of the HZAO thin films at a frequency of 50 Hz at room temperature in light-tight condition, with Au/Cr as the top electrode and p^++^-Si as the bottom electrode. Figure 2a shows the P-E curves of the Hf_0.4_Zr_0.4_Al_0.2_O_y_, Hf_0.45_Zr_0.45_Al_0.1_O_y_, Hf_0.475_Zr_0.475_Al_0.05_O_y_ and Hf_0.5_Zr_0.5_O samples; the inset shows the extracted ferroelectric parameters. The Hf_0.475_Zr_0.475_Al_0.05_O_y_ thin film exhibited the strongest ferroelectricity, with a total remnant polarization (2|*Pr*|) of 15.22 μC/cm^2^ and a *Pr/Ec* ratio of 9.06 pF/cm (*Ec* is the coercive field), indicating that the ferroelectricity of the HZO thin film could be enhanced by incorporation of Al with a suitable content (e.g., 5% Al in an HZO thin film).

The capacitance-voltage (*C-V*) curves of the (Au/Cr)/HZAO/p++-Si capacitors were measured by an Agilent 4284A precision LCR meter. Figure 2b shows the *C-V* curves of the HZAO thin films with different Al contents under 5 kHz frequency. A sharp capacitance peak can be observed around 0 V; the higher peak indicates stronger ferroelectricity and a negative-capacitance effect [29]. For the conventional (Cr/Au)/HfO_2_/p-Si MOS capacitor in Figure 2c, we did not observe a capacitance peak, which would exclude the impact of stresses or high frequency signals. The Hf_0.475_Zr_0.475_Al_0.05_O_y_ thin film exhibited the highest capacitance peak, which was consistent with the *P-E* measurement result, indicating that the strongest ferroelectricity was for the Hf_0.475_Zr_0.475_Al_0.05_O_y_ thin film. The peaks indicate that the single layer HZAO film had both positive- and negative-capacitance characteristics and was able to achieve capacitance-matching by itself without another positive capacitance layer.

**Figure 3:**
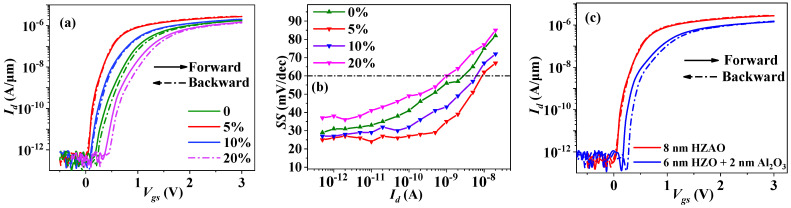
(**a**) Transfer curves and (**b**) *SS-I_d_* curves for the HZAO MoS_2_ NCFETs with different Al contents (annealing temperature: 600 °C); (**c**) Transfer curves of HZAO NCFETs and HZO-Al_2_O_3_ NCFETs.

The drain current vs. gate-source voltage (*I_d_-V_gs_*) curves of the transistors were measured using a Keithley 4200 SCS semiconductor parameter analyzer in a light-tight and electrically-shielded probe station in an atmospheric pressure environment at room temperature, as shown in Figure 3a. The sweeping range of *V_gs_* was from −1 V to 3 V, with a sweeping rate of 0.2 V/s and drain-source voltage (*V_ds_*) fixed at 50 mV. The *SS-I_d_* relations were extracted from the transfer curves and are shown in Figure 3b.

Table 1 lists the off-current (*I_off_*), on-current (*I_on_*), mobility, *SS* and hysteresis of the HZAO NCFETs with different Al contents, which were extracted from their relevant transfer curves.

From Figure 3 and Table 1, it can be seen that the HZAO NCFETs with different Al contents exhibited similar *I_off_* and mobility, indicating that the insulation integrity of the HfZrO thin film was not influenced by the incorporation of Al atoms. It is especially worth noting that the Hf_0.475_Zr_0.475_Al_0.05_O*_y_* NCFET (Al content of 5%) exhibited the lowest *SS* (22.3 mV/dec) over almost four orders of *I_d_* magnitude, the largest *I_on_* (2.8 μA/μm) and the smallest hysteresis (~10 mV) among all the samples, i.e., a suitable Al-doped concentration was determined to be 5%, at which a high switching speed from the off-state to the on-state can be obtained. In addition, comparison of electrical properties between the single-layer HZAO and traditional multi-layer gate stack NCFETs was performed, as shown in Figure 3c and Table 2. For the same thickness of the gate stack, the former exhibited better electrical parameters than the latter. According to the formulas of *SS* = $\left(ln10\right)\times \left(\frac{k_{B}T}{q}\right)\times \left(1+\frac{C_{s}+C_{it}}{C_{ox}}\right)$ and $C_{it}=q^{2}D_{it}$ [30], the interface-state density between MoS_2_ and positive HZAO was determined to be in a range of (2.75~2.94) × 10^12^ eV^−1^cm^−2^, which would not lead to relatively large hysteresis.

### 3.2. Optimization of Annealing Temperature

**Figure 4:**
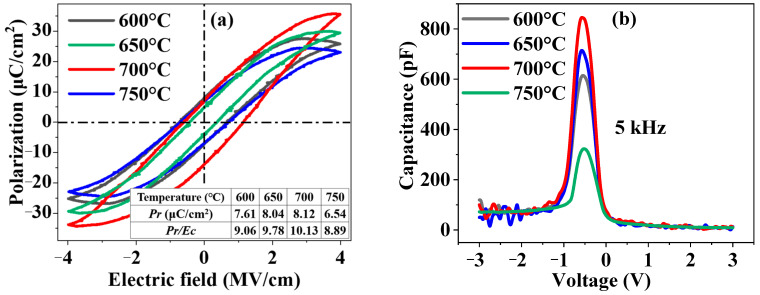
(**a**) *P-E* curves measured at 50 Hz and (**b**) *C-V* curves measured at 5 kHz of (Au/Cr)/Hf_0.475_Zr_0.475_Al_0.05_O*_y_*/p^++^-Si capacitors with different annealing temperatures (Al ratio: 5%).

Based on the above results, the Hf_0.475_Zr_0.475_Al_0.05_O*_y_* thin film was selected to investigate its optimum annealing temperature. The *P-E* curves of the Hf_0.475_Zr_0.475_Al_0.05_O*_y_* thin films annealed at 600 °C, 650 °C, 700 °C and 750 °C, respectively, are shown in Figure 4a. The 700 °C annealed Hf_0.475_Zr_0.475_Al_0.05_O*_y_* thin film exhibited the strongest ferroelectricity, with |2*P_r_*| of 15.76 μC/cm^2^ and a *P_r_*/*E_c_* ratio of 9.27 pF/cm.

The *C-V* curves of the (Au/Cr)/Hf_0.475_Zr_0.475_Al_0.05_O*_y_*/p^++^-Si capacitors for different annealing temperatures were measured at 5 kHz, as shown in Figure 4b. The Hf_0.475_Zr_0.475_Al_0.05_O*_y_* thin film annealed at 700 °C exhibited the highest capacitance peak, which was consistent with the *P-E* measurement result, supporting it exhibiting the strongest ferroelectricity, as shown in Figure 4a.

**Figure 5:**
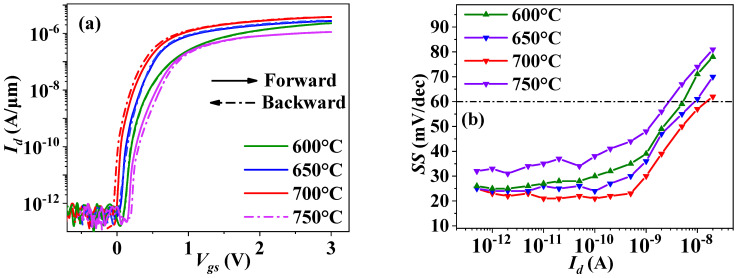
(**a**) Transfer curves and (**b**) *SS-I_d_* curves for the Hf_0.475_Zr_0.475_Al_0.05_O_y_ NCFETs with different annealing temperatures (Al ratio: 5%).

The *I_d_-V_gs_* curves and the *SS-I_d_* curves of the Hf_0.475_Zr_0.475_Al_0.05_O*_y_* NCFETs for different annealing temperatures are shown in Figure 5a,b, respectively; the relevant electrical parameters extracted from their transfer curves are listed in Table 2.

From Figure 5 and Table 3, it can be observed that the Hf_0.475_Zr_0.475_Al_0.05_O_y_ NCFET annealed at 700 °C exhibited the lowest *SS* (20.4 mV/dec) by almost four orders of *I_d_* magnitude with a very small anticlockwise hysteresis of −5~−10 mV, attributed to its enhanced ferroelectricity (large *P_r_/E_c_* ratio and |2*P_r_*|), as shown in Figure 4a, resulting in a large ferroelectric capacitance (|*C_FE_*|) and, thus, a better capacitance match [31,32]. However, the 650 °C-annealed NCFET exhibited the second lowest *SS* (22.3 mV/dec) without hysteresis. This is because the hysteresis of the MoS_2_ NCFETs is influenced by both the interface states and the NC effect. The hysteresis caused by interfacial defaults is clockwise [33] and an anticlockwise hysteresis is introduced by the NC effect of the ferroelectric thin film [1]. So, the total hysteresis for the MoS_2_ NCFETs, with HZAO as the gate dielectric, is a sum of the two kinds of hysteresis [34]. Assuming a consistent interface property for the MoS_2_ NCFETs annealed at 650 °C and 700 °C, i.e., that the two samples have the same clockwise hysteresis, the total hysteresis depends on their anticlockwise hysteresis. For the 650 °C annealed Hf_0.475_Zr_0.475_Al_0.05_O*_y_* NCFET, its clockwise hysteresis just counteracted its anticlockwise hysteresis, resulting in hysteresis-free behavior. For the 700 °C-annealed Hf_0.475_Zr_0.475_Al_0.05_O*_y_* NCFET, its anticlockwise hysteresis was slightly larger than its clockwise hysteresis, resulting in a total anticlockwise hysteresis of −5~−10 mV (almost hysteresis-free behavior). Therefore, it is suggested that a reasonable range for the annealing temperature is 650 °C~700 °C.

**Figure 6:**
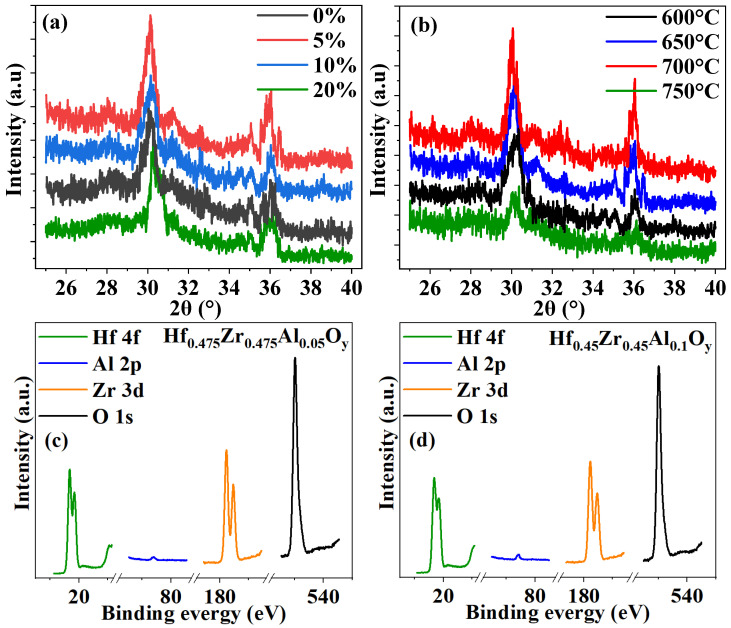
XRD patterns of (**a**) HZAO thin films with different Al contents and (**b**) Hf_0.475_Zr_0.475_Al_0.05_O_y_ thin films annealed at 600 °C, 650 °C, 700 °C and 750 °C, respectively; Hf 4f, Zr 3d, Al 3p and Q 1s XPS spectra of (**c**) Hf_0.475_Zr_0.475_Al_0.05_O*_y_* thin film and (**d**) Hf_0.45_Zr_0.45_Al_0.1_O*_y_* thin film.

To support the electrical results, X-ray diffraction (XRD) and X-ray photoelectron spectroscopy (XPS) measurements were undertaken on the HZAO thin films for different Al contents and annealing temperatures to investigate their crystal structure. Figure 6a,b show the XRD patterns of the HZAO thin films with different Al contents and annealing temperatures, respectively. The diffraction peaks occurring at around 30.5° and 36° are attributed to the orthorhombic phase [16,35,36]; a higher peak implies an enhanced orthorhombic phase and, thus, stronger ferroelectricity [19,37]. The 5%Al content and 700 °C annealed HZAO thin films exhibited the highest diffraction peaks, indicating the strongest ferroelectricity, which is consistent with the *P-E* measurement results shown in Figure 2a and Figure 4a.

Figure 6c,d show the Hf 4f, Zr 3d, Al 3p and Q 1s XPS spectra of the Hf_0.475_Zr_0.475_Al_0.05_O*_y_* and Hf_0.45_Zr_0.45_Al_0.1_O*_y_* thin films, respectively. The percentage of the Al element in the two thin films was 2.62% and 5.33%, respectively, based on calculation of the Al 3p/O1s peak-area ratio. Similarly, the Hf 4f/O 1s and Zr 3d/O 1s ratios were calculated to be 48.23% and 46.53%, for the Hf_0.475_Zr_0.475_Al_0.05_O*_y_* sample, and 45.36% and 43.98%, for the Hf_0.45_Zr_0.45_Al_0.1_O*_y_* sample. So, their chemical formulae were Hf_0.4823_Zr_0.4653_Al_0.0524_O*_y_* and Hf_0.4536_Zr_0.4398_Al_0.1066_O*_y_*, respectively, which is consistent with the atom ratios of the ALD-yielded HZAO thin films, confirming the validity of the electrical results.

## 4. Conclusions

In this investigation, back-gated MoS_2_ NCFETs with a single HZAO layer as the gate dielectric were successfully fabricated. The effects of Al content and annealing temperature on the device performances were investigated. It was found that the 700°C-annealed Hf_0.475_Zr_0.475_Al_0.05_O*_y_* (5%Al content) NCFET exhibited the lowest *SS* (~20 mV/dec), the highest *I_on_/I_off_* (9.1~9.8 × 10^6^) and almost hysteresis-free behavior, attributed to its strong ferroelectricity and NC effects. The 650°C-annealed Hf_0.475_Zr_0.475_Al_0.05_O*_y_* NCFET exhibited better comprehensive performances, including low *SS* (~22 mV/dec), no hysteresis and a high *I_on_/I_off_* ratio [(8.8~9.5) × 10^6^], attributed to its second strongest ferroelectricity and the excellent balance between anticlockwise hysteresis from the NC effect and clockwise hysteresis from the HZAO/MoS_2_ interface.

XRD, *C-V* and *P-E* measurements were used to confirm the ferroelectricity of the Hf_0.5−0.5*x*_Zr_0.5−0.5*x*_Al*_x_*O*_y_* thin films with different Al contents and annealed at different temperatures, indicating strong ferroelectricity for the Hf_0.475_Zr_0.475_Al_0.05_O*_y_* samples annealed at 650~700 °C, with higher diffraction peaks of the orthorhombic phase and *C-V* peaks relevant to the NC effect, and large *P_r_*. Compared to single HZO layer and gate-stacked NCFETs, the single HZAO layer gate-dielectric NCFET has significant potential for small-scale and low-power devices due to its excellent sub-threshold properties and simplified preparation process.

## Figures and Tables

**Table 1:** Electrical parameters of HZAO NCFETs with different Al contents extracted from their transfer curves (annealing temperature of 600 °C).

Sample	Hf_0.4_Zr_0.4_Al_0.2_O*_y_*	Hf_0.45_Zr_0.45_Al_0.1_O*_y_*	Hf_0.475_Zr_0.475_Al_0.05_O*_y_*	Hf_0.5_Zr_0.5_O*_y_*
*I_off_* (pA/μm)	0.3~1	0.3~1	0.3~1	0.3~1
*I_on_* (μA/μm)	1.1~1.2	1.9~2.2	2.5~2.8	1.5~1.7
*Mobility* (cm^2^/Vs)	35.8~38.4	35.6~38.5	36.2~38.7	35.5~37.8
*SS* (mV/dec)	35.0~45.1	24.4~26.9	22.3~25.6	28.8~40.6
*Hysteresis* (mV)	30~50	15~20	5~10	20~30
*V_TH_ (V)*	0.55~0.60	0.15~0.20	0.10~0.15	0.40~0.45

**Table 2:** Electrical parameters of the HZAO NCFETs and HZO-Al_2_O_3_ NCFETs.

Gate Stack	8 nm HZAO	6 nm HZO +2 nm Al_2_O_3_
*I_off_* (pA/μm)	0.3~1	0.3~1
*I_on_* (μA/μm)	2.5~2.8	1.4~1.6
*Mobility* (cm^2^/Vs)	36.2~38.7	35.8~37.5
*SS* (mV/dec)	22.3~25.6	33.5~41.4
*Hysteresis (mV)*	5~10	40~50
*V_TH_ (V)*	0.10~0.15	0.25~0.30

**Table 3:** Zr_0.475_Al_0.05_O*_y_* NCFETs with different annealing temperatures (*T*) extracted from their transfer curves.

*T* (°C)	600	650	700	750
*I_off_* (pA/μm)	0.3~1	0.3~1	0.3~1	0.3~1
*I_on_* (μA/μm)	2.5~2.8	2.4~2.6	2.5~2.8	1.5~1.7
*Mobility* (cm^2^/Vs)	36.2~38.7	36.6~38.3	36.4~38.7	35.2~37.4
*SS* (mV/dec)	22.3~25.6	22.3~25.6	20.4~23.1	30.3~40.7
*Hysteresis (mV)*	5~10	0	−5~−10	40~50
*V_TH_ (V)*	0.10~0.15	0.05~0.10	0~0.05	0.20~0.25

## Data Availability

The data that support the findings of this study are available from the corresponding author upon reasonable request.
